# Supplementary material for: Resolving the full spectrum of human genome variation using Linked-Reads
Source: Genome Res. 2019 Apr;29(4):635–45. doi: 10.1101/gr.234443.118 (PMC6442396; doi:10.1101/gr.234443.118)
Supplement: Supplemental Material [file supp_gr.234443.118_Supplemental_longranger-2.2.zip › longranger-2.2.tar.bz2!/lr2.2-public/lib/stan/cmdstan/stan/doc/api/html/functions__grammar__def_8hpp.html]

Stan: src/stan/gm/grammars/functions\_grammar\_def.hpp File Reference


|  |  |
| --- | --- |
|  | Stan  2.5.0  probability, sampling & optimization |


- Main Page
- Related Pages
- Namespaces
- Classes
- Files

- File List
- File Members

All Classes Namespaces Files Functions Variables Typedefs Enumerations Enumerator Friends Macros Pages

- src
- stan
- gm
- grammars

Classes |
Namespaces |
Functions |
Variables

functions\_grammar\_def.hpp File Reference

`#include <set>`  
`#include <utility>`  
`#include <vector>`  
`#include <boost/spirit/include/qi.hpp>`  
`#include <boost/lexical_cast.hpp>`  
`#include <boost/fusion/include/adapt_struct.hpp>`  
`#include <boost/fusion/include/std_pair.hpp>`  
`#include <boost/config/warning_disable.hpp>`  
`#include <boost/spirit/include/qi_numeric.hpp>`  
`#include <boost/spirit/include/phoenix_core.hpp>`  
`#include <boost/spirit/include/phoenix_function.hpp>`  
`#include <boost/spirit/include/phoenix_fusion.hpp>`  
`#include <boost/spirit/include/phoenix_object.hpp>`  
`#include <boost/spirit/include/phoenix_operator.hpp>`  
`#include <boost/spirit/include/phoenix_stl.hpp>`  
`#include <boost/spirit/include/support_multi_pass.hpp>`  
`#include <boost/tuple/tuple.hpp>`  
`#include <boost/variant/apply_visitor.hpp>`  
`#include <boost/variant/recursive_variant.hpp>`  
`#include <boost/spirit/include/version.hpp>`  
`#include <boost/spirit/include/support_line_pos_iterator.hpp>`  
`#include <stan/gm/ast.hpp>`  
`#include <stan/gm/grammars/functions_grammar.hpp>`  
`#include <stan/gm/grammars/statement_grammar.hpp>`  
`#include <stan/gm/grammars/whitespace_grammar.hpp>`

Go to the source code of this file.

|  |  |
| --- | --- |
| Classes | |
| struct | stan::gm::validate\_non\_void\_arg\_function |
|  | |
| struct | stan::gm::validate\_non\_void\_arg\_function::result< T1, T2, T3 > |
|  | |
| struct | stan::gm::set\_void\_function |
|  | |
| struct | stan::gm::set\_void\_function::result< T1, T2, T3, T4 > |
|  | |
| struct | stan::gm::set\_allows\_sampling\_origin |
|  | |
| struct | stan::gm::set\_allows\_sampling\_origin::result< T1, T2, T3 > |
|  | |
| struct | stan::gm::validate\_declarations |
|  | |
| struct | stan::gm::validate\_declarations::result< T1, T2, T3, T4 > |
|  | |
| struct | stan::gm::add\_function\_signature |
|  | |
| struct | stan::gm::add\_function\_signature::result< T1, T2, T3, T4, T5 > |
|  | |
| struct | stan::gm::validate\_return\_type |
|  | |
| struct | stan::gm::validate\_return\_type::result< T1, T2, T3 > |
|  | |
| struct | stan::gm::scope\_lp |
|  | |
| struct | stan::gm::scope\_lp::result< T1 > |
|  | |
| struct | stan::gm::unscope\_variables |
|  | |
| struct | stan::gm::unscope\_variables::result< T1, T2 > |
|  | |
| struct | stan::gm::add\_fun\_var |
|  | |
| struct | stan::gm::add\_fun\_var::result< T1, T2, T3, T4 > |
|  | |

|  |  |
| --- | --- |
| Namespaces | |
|  | stan |
|  | Probability, optimization and sampling library. |
|  | |
|  | stan::gm |
|  | Directed graphical model compiler. |
|  | |

|  |  |
| --- | --- |
| Functions | |
|  | BOOST\_FUSION\_ADAPT\_STRUCT (stan::gm::function\_decl\_def,(stan::gm::expr\_type, return\_type\_)(std::string, name\_)(std::vector< stan::gm::arg\_decl >, arg\_decls\_)(stan::gm::statement, body\_)) |
|  | |
|  | BOOST\_FUSION\_ADAPT\_STRUCT (stan::gm::arg\_decl,(stan::gm::expr\_type, arg\_type\_)(std::string, name\_)(stan::gm::statement, body\_)) |
|  | |

|  |  |
| --- | --- |
| Variables | |
| boost::phoenix::function  < validate\_non\_void\_arg\_function > | stan::gm::validate\_non\_void\_arg\_f |
|  | |
| boost::phoenix::function  < set\_void\_function > | stan::gm::set\_void\_function\_f |
|  | |
| boost::phoenix::function  < set\_allows\_sampling\_origin > | stan::gm::set\_allows\_sampling\_origin\_f |
|  | |
| boost::phoenix::function  < validate\_declarations > | stan::gm::validate\_declarations\_f |
|  | |
| boost::phoenix::function  < add\_function\_signature > | stan::gm::add\_function\_signature\_f |
|  | |
| boost::phoenix::function  < validate\_return\_type > | stan::gm::validate\_return\_type\_f |
|  | |
| boost::phoenix::function  < scope\_lp > | stan::gm::scope\_lp\_f |
|  | |
| boost::phoenix::function  < unscope\_variables > | stan::gm::unscope\_variables\_f |
|  | |
| boost::phoenix::function  < add\_fun\_var > | stan::gm::add\_fun\_var\_f |
|  | |

## Function Documentation

|  |  |  |  |
| --- | --- | --- | --- |
| BOOST\_FUSION\_ADAPT\_STRUCT | ( | stan::gm::function\_decl\_def | , |
|  |  | (stan::gm::expr\_type, return\_type\_)(std::string, name\_)(std::vector< stan::gm::arg\_decl >, arg\_decls\_)(stan::gm::statement, body\_) |  |
|  | ) |  |  |

|  |  |  |  |
| --- | --- | --- | --- |
| BOOST\_FUSION\_ADAPT\_STRUCT | ( | stan::gm::arg\_decl | , |
|  |  | (stan::gm::expr\_type, arg\_type\_)(std::string, name\_)(stan::gm::statement, body\_) |  |
|  | ) |  |  |


---

[ Stan Home Page ]

*© 2011–2014,
Stan Development Team.*
